# Atypical otolaryngologic manifestations of COVID-19: a review

**DOI:** 10.1186/s43163-021-00075-z

**Published:** 2021-01-21

**Authors:** Mohamed Zahran, Rania Ghazy, Omar Ahmed, Ahmed Youssef

**Affiliations:** 1 Department of Otolaryngology, Alexandria University School of Medicine, Champllion Street, El-Azareeta, Alexandria, Egypt; 2 East Kent University Hospital, National Health Trust, London, UK

**Keywords:** Corona virus, COVID-19, SARS-CoV-2, Otolaryngology

## Abstract

**Background:**

COVID-19 pandemic caused by SARS-CoV-2 started in China in late 2019. Clinical features include fever, cough, dyspnea, body aches, and gastrointestinal symptoms. Some COVID-19-positive patients presented with unusual manifestations such as olfactory dysfunction, parotitis, or cervical lymphadenopathy.

**Main body:**

Since many patients are diagnosed with SARS-CoV-2, the need for an up to date review of the atypical ENT presentations of COVID-19 is mandatory. Articles from PubMed and Google searches were reviewed and the atypical presentations in otolaryngology were presented.

**Conclusions:**

It is crucial for ENT physicians to have high index of suspicion to identify those COVID 19 patients with atypical presentations. This facilitates early case isolation to eliminate viral spread across the community.

## Background

COVID-19 pandemic caused by SARS-CoV-2 started in China in late 2019 [1]. The main route of viral spread is through aerosol transmissions. As of today, 26 million persons got infected and 880,000 died of the disease [2].

COVID-19 has wide spectrum of clinical features starting from mild disease to severe illness. Incubation period ranges from 2 to 14 days. Common clinical features include fever, cough, rhinorrhea, dyspnea, and body ache. Other less common manifestations include nausea, vomiting, or diarrhea [3, 4].

## Methods

Source information was sought via PubMed and Google searches for “(coronavirus or COVID) and atypical presentations.” Online content was specifically sought because of the very recent nature of many source documents.

### Review methods

All sources presenting objective evidence related to the topic were reviewed and filtered.

### Ethical considerations

The study was approved by The Hospital Ethics Committee and informed consent was not mandatory.

## Results

The spectrum of the atypical presentations of COVID-19 in otolaryngology is summarized in Table 1 and presented in details in the following sections.

**Table 1:** Atypical manifestation of COVID-19 in otolaryngology

Organ/subsite	Atypical manifestations
Ear	1. Acute otitis media 2.SNHL
Nose	Olfactory dysfunction
Oral cavity and oropharynx	1.Taste dysfunction 2.Vesiculobullous lesions 3.Ulcers and blisters 4.Strawberry tongue (Kawasaki-like)
Lymph nodes	Cervical lymphadenopathy
Parotid gland	1. Acute parotitis 2. Parotitis-like disease
Thyroid gland	Subacute thyroiditis

### COVID-19-induced acute otitis media

Fidan [5] reported a case of COVID-19-induced acute otitis media (AOM) in 35-year-old female. The patient initially presented with otalgia and tinnitus and there were no other symptoms of COVID-19. Otoscopy revealed right red bulging tympanic membrane. Audiologic investigations demonstrated right conductive hearing loss and type B tympanometry. Due to the incidental finding of mild rhonchi and the current pandemic, chest X-ray and RT-PCR were done. The result came as COVID-19 positive.

### Sensorineural hearing loss (auditory dysfunction)

The first case of COVID-19-induced sensorineural hearing loss (SNHL) was reported in Thailand in an elderly female. The patient recovered from COVID-19 infection; however, the hearing loss did not improve [6].

The effect of COVID-19 is an interesting issue in audiology. Several viral infections can directly damage inner ear structures causing hearing loss. Typically, virus-induced hearing loss is sensorineural (SNHL). The proposed mechanisms involved in virus-induced SNHL include direct damage to inner ear or indirect damage through induction of autoimmune response [7, 8]. Previous studies on other coronavirus infection revealed brainstem involvement and suggested possible affection of the auditory neural pathway [6].

A recent study of the effect of COVID-19 on the inner ear structures was done. The author compared between asymptomatic COVID-19 PCR-positive cases and control subjects. The persons enrolled in the study were 20–50 years to rule out any age-related hearing loss. Exclusion criteria were symptomatic COVID-19 patients and past history of hearing loss. The high-frequency pure tone thresholds and TEOAE amplitudes were significantly worse in COVID-19 patients [9].

Cure et al. [10] suggested the possible mechanism of COVID-19-induced SNHL. The entry point of COVID-19 is the lung and gets inside the cell by penetrating to the angiotensin-converting enzyme 2 (ACE2) [11]. COVID-19 binds to the beta chain of hemoglobin, transported by blood, and can penetrate all tissues with ACE2 [12]. Binding of COVID-19 to ACE2 depends on cytosolic pH. Heavy infection in the elderly is facilitated by their low cytosolic pH [11, 13]. The brain, medulla oblongata and the hearing center in the temporal lobe all have plenty of ACE2 [14]. Viral-induced cytokine release leads to irreversible hearing loss (oxidative stress). Oxygen release from the infected RBCs is low leading to tissue hypoxia and further cellular damage. There is ACE2 in vascular smooth muscles and COVID-19 is known to increase thrombosis risk. The elderly have preexisting risk factors for thrombosis (hypertension, diabetes mellitus, atherosclerosis) and this risk is amplified by COVID-19. The hearing loss is probably irreversible as it involves damage to central neuroauditory structures [10].

### Smell and taste dysfunction

Many physicians reported anosmia, with or without dysgeusia in patients with severe COVID-19 infection. The AAO-HNS and the British Association of Otorhinolaryngology are now recommending these symptoms to be added to the list of primary screening symptoms for COVID-19.

A multi-center European study done by Lechien et al. [15] reported 357 patients with COVID-19 olfactory dysfunction. In a study by Klopfenstein et al., 54 of 114 patients (47%) with confirmed COVID-19 reported anosmia. Anosmia began 4.4 ± 1.9 days after infection onset. The mean duration of anosmia was 8.9 ± 6.3 days and 98% of patients recovered within 28 days. Eighty-five percent had dysgeusia and 28% presented with pneumonia [16].

The concept of anosmia after viral infection is known as post-infectious/post-viral olfactory loss. Different kinds of viruses can induce olfactory loss, including COVID-19 [17]. The exact mechanism is not fully understood. Viral infection of the olfactory system by neurotropic viruses is possible explanation [18]. However, this was not confirmed by neuroimaging studies or histopathological tissue specimens of the olfactory neuroepithelium [19].

### Cervical lymphadenopathy

Presence of cervical lymphadenopathy or strawberry tongue in children can be part of Kawasaki-like disease. There is growing number of children reported with COVID-19-induced Kawasaki-like disease. Kawasaki disease is an acute self-limiting vasculitis of the medium size blood vessels and almost exclusively affects children. Kawasaki disease was first reported by Tomisaku Kawasaki in Japan [20]. The exact cause of Kawasaki disease remains unknown. The most accepted theory suggests an aberrant response of the immune system to unknown pathogens in genetically susceptible patients [21].

The American Heart Association diagnostic criteria include (fever for ≥ 5 days plus four or more clinical criteria, including bilateral bulbar non-exudative conjunctivitis, changes of the lips or oral cavity, non-suppurative lateral cervical lymphadenopathy, polymorphic rash, erythema of the palms and soles, firm induration of the hands or feet, or both) [22]. Early recognition of this serious disease facilitates appropriate referral to the pediatrician.

### Oral manifestations in COVID-19

Many viruses including COVID-19 can present with exanthematous lesions as oral ulcers or blisters. The pathogenesis is similar to aphthous fever, hand, foot, and mouth disease, and herpetic gingivostomatitis [23]. Painful palatal ulcers were reported in two male patients 56 and 58 years old. There were multiple small ulcers with an erythematous halo on the hard palate with unilateral distribution. The lesions were similar to a herpetic recurrent stomatitis; although, the patients had no past history of herpetic infection. Complete recovery occurred within 10 days. Another COVID-19 patient presented with inner lip blisters and desquamative gingivitis. Improvement occurred within 3 days of oral steroids [24]. Similar study reported occurrence of irregular asymptomatic tongue ulcer in COVID-19 positive female [25].

### Parotitis and parotitis-like disease

Many viruses as rubella, influenza, and HIV have predilection for salivary glands including the parotid [26]. Parotitis was reported to occur in association with COVID-19 [27]. Authors from France reported parotitis-like manifestations in three COVID-19-positive patients. They validated the occurrence of intraparotid lymphadenitis by MRI findings. Complete recovery of parotitis occurred within few days [28].

### Subacute thyroiditis in COVID-19

The pathogenesis of subacute thyroiditis in the setting of viral infections like COVID-19 is unclear. The disease may be directly related the viral infection or indirectly as a result of post- viral inflammatory response in genetically susceptible persons [29].

Authors reported a case of subacute thyroiditis in an 18-year-old female with COVID-19. Clinical manifestations included pain, tenderness, and enlargement in the thyroid gland. No history of upper respiratory tract infection was present. Laboratory work up showed elevated T3 and T4. Other markers of inflammation as ESR, CRP, and leucocytic count were also elevated. Thyroid US revealed multiple diffuse hypoechoic areas. Complete recovery of inflammation and improvement of thyroid function occurred within 2 weeks of prednisone treatment [30].

## Conclusion

It is crucial for ENT physicians to have high index of suspicion to identify those COVID-19 patients with atypical presentations. This facilitates early case isolation to eliminate viral spread across the community.

## Data Availability

Not applicable

## Abbreviations

AAO-HNS: American academy of otolaryngology**-**head and neck surgery
COVID-19: Corona virus disease
CRP: C-reactive protein
ESR: Erythrocyte sedimentation rate
ENT: Ear, nose, throat
MRI: Magnetic resonance imaging
ORL: Otorhinolaryngology
RT-PCR: Real-time polymerase chain reaction
RBCs: Red blood cells
SNHL: Sensorineural hearing loss
SARS-CoV-2: Severe acute respiratory syndrome caused by corona virus 2
TEOAE: Transient evoked otoacoustic emission
